# The Effect of *Bifidobacterium animalis* subsp. *lactis* MN-Gup on Glucose Metabolism, Gut Microbiota, and Their Metabolites in Type 2 Diabetic Mice

**DOI:** 10.3390/nu16111691

**Published:** 2024-05-29

**Authors:** Chao Zhang, Bing Fang, Nana Zhang, Qi Zhang, Tianjiao Niu, Liang Zhao, Erna Sun, Jian Wang, Ran Xiao, Jingjing He, Shusen Li, Juan Chen, Jie Guo, Wei Xiong, Ran Wang

**Affiliations:** 1 Department of Nutrition and Health, Key Laboratory of Functional Dairy, Co-Constructed by Ministry of Education and Beijing Government, China Agricultural University, Beijing 100193, China; 2 Food Laboratory of Zhongyuan, Luohe 462300, China; 3 Research Center for Probiotics, China Agricultural University, Beijing 100193, China; 4 Mengniu Hi-Tech Dairy Product Beijing Co., Ltd., Beijing 101100, China; 5 Beijing Laboratory of Food Quality and Safety, College of Food Science and Nutritional Engineering, China Agricultural University, Beijing 100083, China

**Keywords:** type 2 diabetes mellitus, MN-Gup, probiotics, glucagon-like peptide-1, short-chain fatty acids

## Abstract

Probiotics have garnered increasing attention as a potential therapeutic approach for type 2 diabetes mellitus (T2DM). Previous studies have confirmed that *Bifidobacterium animalis* subsp. *lactis* MN-Gup (MN-Gup) could stimulate the secretion of glucagon-like peptide-1 (GLP-1) in NCI-H716 cells, but whether MN-Gup has a hypoglycemic effect on T2DM in vivo remains unclear. In this study, a T2DM mouse model was constructed, with a high-fat diet and streptozotocin in mice, to investigate the effect of MN-Gup on diabetes. Then, different doses of MN-Gup (2 × 10^9^ CFU/kg, 1 × 10^10^ CFU/kg) were gavaged for 6 weeks to investigate the effect of MN-Gup on glucose metabolism and its potential mechanisms. The results showed that a high-dose of MN-Gup significantly reduced the fasting blood glucose (FBG) levels and homeostasis model assessment-insulin resistance (HOMA-IR) of T2DM mice compared to the other groups. In addition, there were significant increases in the short-chain fatty acids (SCFAs), especially acetate, and GLP-1 levels in the MN-Gup group. MN-Gup increased the relative abundance of *Bifidobacterium* and decreased the number of *Escherichia-Shigella* and *Staphylococcus*. Moreover, the correlation analysis revealed that *Bifidobacterium* demonstrated a significant positive correlation with GLP-1 and a negative correlation with the incremental AUC. In summary, this study demonstrates that *Bifidobacterium animalis* subsp. *lactis* MN-Gup has significant hypoglycemic effects in T2DM mice and can modulate the gut microbiota, promoting the secretion of SCFAs and GLP-1.

## 1. Introduction

Diabetes mellitus is a metabolic disease characterized by chronic hyperglycemia, in which 90% to 95% of patients have type 2 diabetes (T2DM) [1]. The International Diabetes Federation (IDF) reported that the global prevalence of T2DM has reached 10.5% and is expected to increase to 12.2% by 2045 [2]. Previous reports have shown that the gut microbiota is considered an important factor affecting the pathogenesis of T2DM [3]. The gut microbiota is involved in the process of energy metabolism. It can utilize undigested and unabsorbed ingredients to participate in the metabolism of carbohydrates, proteins, choline, and primary bile acids. in addition, it produces bioactive metabolites such as short-chain fatty acids (SCFAs) and branched-chain amino acids, etc. These metabolites play a vital role in the development of diabetes [4].

Recently, the application of probiotics has been considered as a potential method for the treatment of T2DM [5]. For example, *Lactobacillus* MCC2759 and MCC2760 have been demonstrated to improve oral glucose tolerance and insulin levels in T2DM rats [6]. Most importantly, probiotics have been shown to improve T2DM symptoms through gut microbiota and the glucagon-like peptide-1 (GLP-1) [7]. *Lactobacillus* paracasei JY062 has the potential to prevent and alleviate T2DM by altering the gut microbiota structure, enhancing the intestinal barrier function, and promoting GLP-1 and PYY secretion [8]. Thus, probiotics might contribute to the development of insulin resistance by altering the gut microbiota composition and the GLP-1 release.

*Bifidobacterium animalis* subsp. *lactis* MN-Gup (MN-Gup, CGMCC No. 15578) is a strain derived from aerospace mutation screenings of *Bifidobacterium animalis* subsp. *lactis* BB-11 (CGMCC No. 14056), which was transported by the Shenzhou-11 re-entry spacecraft [9]. Previous studies have confirmed that MN-Gup has the effect of improving obesity and constipation [9,10]. MN-Gup can also enrich bacterial genera such as *Ruminiclostridium*, *Blautia*, *Rumminococcaceae*, and *Ruminoccaceae_UCG-002* [9,10]. Most importantly, it has been previously demonstrated that MN-Gup stimulates NCI-H716 cells to secrete GLP-1, and MN-Gup was considered to have the potential to treat T2DM [11]. Therefore, in this study, we explored the effects of MN-Gup on glucose metabolism in diabetic mice and its potential mechanism.

## 2. Methods

### 2.1. Animals and Treatment

**Figure 1:**
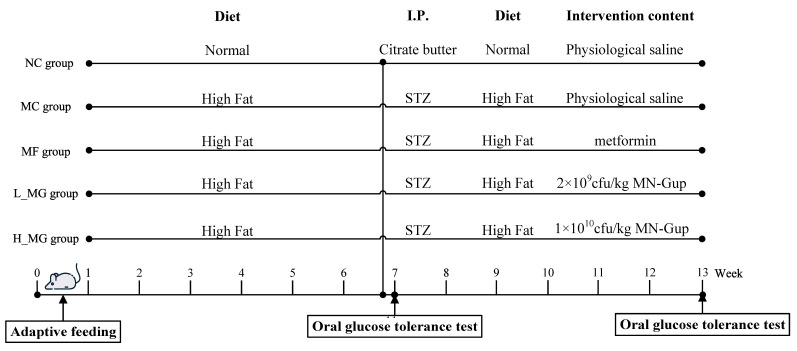
Experimental schedule. STZ: streptozotocin.

Thirty 5-week-old C57BL/6J male mice, bred under Specific Pathogen-Free (SPF) conditions, were procured from Beijing Weitong Lihua Laboratory Animal Technology Co. Ltd. (Beijing, China). They were then housed in a laboratory animal facility with SPF-grade conditions at Pony Testing International Group Co., Ltd. (Beijing, China). The mice were maintained under a standard light/dark cycle (12 h light/12 h dark) with ad libitum access to both water and food. This study was conducted following approval from the institutional animal ethics committee (approval number: PONY-2023-FL-21), ensuring compliance with scientific standards and ethical guidelines. After a week-long acclimatization period, each mouse was assigned a unique identifier. These identifiers were then inputted into a randomization software program (R version 4.4.0), which allocated the animals randomly to their respective treatment groups. Group assignments were performed by a researcher (JH), who was not involved in the subsequent stages of the experiment, and during the allocation phase, the experimenters were kept blinded. Each group consisted of six mice, resulting in a total of five groups (Figure 1). Each cage, housing six male mice, served as the experimental unit, with treatments randomized and applied at the cage level to ensure that all animals within a cage received the same treatment. To mitigate potential interactions, the cages were adequately spaced to minimize visual and auditory interactions, and regular cleaning and disinfection protocols were followed to maintain the SPF environment. One group of mice was randomly selected as the normal control (NC) group and injected with a sodium citrate buffer (0.05 M, pH = 4.5) on the last day of the sixth week. The remaining four groups of mice were fed with high-fat diets for five weeks and then injected with streptozotocin (STZ, 35 mg/kg, dissolved in 0.05 M sodium citrate buffer, pH = 4.5) through intraperitoneal injection on the same day. Mice exhibiting fasting blood glucose (FBG) levels exceeding 11.1 mmol/L after 3 days of injection were classified as animals with T2DM [12].

The probiotic solid beverage products were produced by Sanhe Fucheng Biological Technology Co. (Langfang, China). The NC group continued to be fed a normal diet and physiological saline. The T2DM mice were divided into four groups, including the diabetic model control group (MC, fed a high-fat diet and physiological saline, serving as the control group), the positive control group (MF, fed a high-fat diet and 120 mg/kg/d metformin), the low-dose MN-Gup group (L-MG, fed a high-fat diet and 2 × 10^9^ CFU/kg MN-Gup), and the high-dose MN-Gup group (H-MG, fed a high-fat diet and 1 × 10^10^ CFU/kg MN-Gup). The body weight of mice was recorded weekly. By the end of the 13th week of intervention, feces were collected following the final gavage. The mice were euthanized the next day, blood samples were drawn, and the serum was prepared. Throughout the experiment, the behavior and health of each animal were closely monitored and recorded to ensure the reliability of the results, account for any potential interactions, detect signs of pain or distress, and establish criteria for humane endpoints to minimize animal sufferings and distress.

### 2.2. The Detection of Glucose Metabolism Indicators

The FBG of mice was measured at weeks 7 and 13. Before the FBG testing, the mice fasted for 8 h. The tail was disinfected with an alcohol swab, and a small incision was made at the tip of the tail vein. The first drop of blood was discarded, and the second drop was used for glucose measurement with a glucometer (Roche Diagnostics, Mannheim, Germany). Simultaneously, blood samples were collected for subsequent indicator testing. The blood samples were centrifuged at 3500 rpm for 15 min, and insulin (Uscn life, Wuhan, China) and GLP-1 (Mesoscale, Rockville, MD, USA) were then detected using ELISA kits. Homeostasis model assessment-insulin resistance (HOMA-IR) was calculated by the following formula [13].

$$HOMA\text{-}IR=\frac{FBG(mmol/L)\times FI(mIU/L)}{22.5}$$

The glucose tolerance of all mice was measured by a glucose meter after modeling (week 7) and before death (week 13), too. Mice were given 2 g/kg·BW glucose solution (40% *w*/*v*) intragastrically after fasting for 16 h. We determined the blood glucose levels in the tail vein blood of mice at 0, 30, 60, 90, and 120 min after gastric irrigation. The results of the Oral Glucose Tolerance Test (OGTT) were quantified as the area under the curve (AUC), which was calculated using GraphPad Prism 9.0 software (San Diego, CA, USA).

### 2.3. Histological Analysis

The colonic and pancreatic tissues of mice were fixed in 4% paraformaldehyde for 24 h. Following fixation, the tissues underwent dehydration, paraffin embedding, and sectioning to obtain thin slices, suitable for microscopic examination. Hematoxylin and eosin (H & E) staining were applied to the sections. Finally, the stained slices were meticulously examined under an optical microscope (DMi8, Leica, Weztlar, Germany) to capture images and analyze histopathological changes.

### 2.4. Gut Microbiota Analysis

Shanghai Majorbio Bio-pharm Technology Co., Ltd. (Shanghai, China) performed the gut microbiota analysis of fresh feces. The DNA of collected colonic content samples was extracted using E.Z.N.A. DNA Kit. Primers 338F (5′-ACTCCTACGGGAGGCAGCAG-3′) and 806R (5′-GGACTACHVGGGTWTCTAAT-3′) were used for PCR amplification of the V3–V4 region of bacterial 16S ribosomal RNA gene. The PCR product was retrieved from a 2% agarose gel and purified using the AxyPrep DNA Gel Extraction Kit from Axygen Biosciences (Union City, CA, USA). Following purification, quantification was carried out utilizing the Quantus™ Fluorometer, manufactured by Promega (Madison, WI, USA). Samples were sequenced on an Illumina MiSeq PE300 platform (Illumina, San Diego, CA, USA). The optimized sequence was obtained by splicing and quality control of sequencing data. Optimizing sequence: 16S rRNA sequences were classified into operational taxonomic units (OTUs), with a threshold of 97% identity, with Uparse (version 7.0.1090).

### 2.5. Short-Chain Fatty Acid Analysis

SCFAs were detected using gas chromatography. Add 50 μL of 50% sulfuric acid to 20 mg of fecal sample, and homogenize the sample for 3 min at 4 °C. Then add 10 μL of internal standard (2.927 mmol/L 2-ethylbutyric acid) and 500 μL of ether, and homogenize for 1 min. Centrifuge at 4600× *g* for 5 min at 4 °C, and collect 350 μL of the supernatant. Repeat this step. Add 500 μL of ether to the pellet, homogenize, and after centrifugation, collect 400 μL of the supernatant. Finally, filter the combined supernatants through a 0.22 μm filter membrane and inject them into brown sample vials for analysis. The specific experimental conditions for gas chromatography were established based on the method developed by Zhang et al. [14].

## 3. Statistical Analysis

The calculation of this study’s sample size was based on the study by Zhou et al. [15]. The outcome measure for determining the sample size is FBG. Assuming a Type I error of 0.05 and a Type II error of 0.10 (β = 90%), the number of mice required to obtain 90% certainty is 4. Considering the uncertainties in animal experiments, the sample size was increased to 6 animals per group.

Data analysis was performed in a double-blinded manner, with both the experimenters (CZ and NZ) and data analyst (BF and RW) being blinded to group assignments. All animals that started the experiment were included in the final analysis. For all data, we assessed the normality of residuals using the Shapiro–Wilk test and confirmed normality through visual inspection of Q-Q plots and histograms. We also evaluated the equality of variances between groups using Levene’s test. Descriptive statistics, presented as mean ± standard deviation (SD), were used to describe the data when they met the assumptions of normality and homogeneity of variances. Group differences were assessed using a one-way analysis of variance (ANOVA), followed by Tukey’s post hoc test for pairwise comparisons. In cases where the data did not meet the assumptions of normality or homogeneity of variances, median (25th percentile, 75th percentile) values were used for description, and group differences were assessed using the Kruskal–Wallis test, followed by Dunn’s post hoc test for pairwise comparisons. Spearman’s correlation analysis was also conducted to explore the relationships between variables. The distance algorithm used for principal coordinates analysis (PCoA) was Bray–Curtis, and inter-group testing was conducted using permutational multivariate analysis of variance (PERMANOVA). The differential abundance of microbial taxa was analyzed using the linear discriminant analysis effect size (LEfSe) method. Taxa with an LDA score ≥ 2.0 and a significance level of *p* < 0.05 were considered statistically significant. Statistical analyses were performed using SPSS 26.0, GraphPad Prism 8, and R version 4.4.0 software packages. Two-sided tests were conducted throughout, with *p* < 0.05 considered statistically significant.

## 4. Results

### 4.1. Effect of MN-Gup on Glucose Metabolism in T2DM Mice

**Figure 2:**
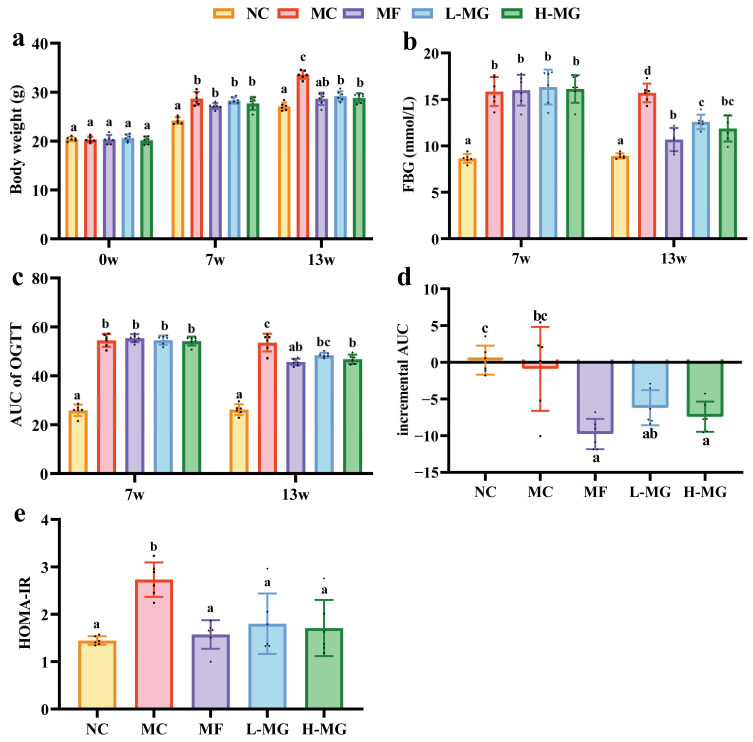
Effect of MN-Gup on glucose metabolism in T2DM mice. (**a**) Body weight of mice at weeks 0, 7 and 13. (**b**) Fasting blood glucose (FBG) levels at weeks 7 and 13. (**c**) Area under curve (AUC) of Oral Glucose Tolerance Test (OGTT). (**d**) The incremental AUC of OGTT at week 13 represents the difference between post-intervention (week 13) and pre-intervention (week 7) results. (**e**) Level of homeostasis model assessment-insulin resistance (HOMA-IR) at week 13. The sample size for each group was 6. Significance analysis was performed using a one-way ANOVA or the Kruskal–Wallis test, with multiple comparisons conducted using Tukey’s test or Dunn’s test, respectively. The letters a, b, c, and d represent the results of multiple comparisons. Different letters signify significant differences between groups (*p* < 0.05), while groups sharing the same letter indicate no significant difference. NC: normal control; MC: diabetic model control; MF: metformin; L-MG: low-dose MN-Gup; H-MG: high-dose MN-Gup.

As shown in Figure 2a, the weight gain of the mice fed high-fat diets was significantly (*p* < 0.05) higher than that of the mice fed normal diets at week 7. Then, STZ was injected at the end of the seventh week. The FBG of the model group was significantly increased compared to that of the NC group (*p* < 0.05), and the FBG was higher than 11.1 mmol/L. This suggested that a mouse model of T2DM was successfully established in the current study. Subsequently, different intervention agents were administered to the corresponding groups, leading to significant alterations in the glucose metabolism in T2DM mice.

As seen in Figure 2b, there were no significant differences observed in the FBG levels among the groups of T2DM mice before the intervention. As the intervention progressed, both the low-dose and high-dose MN-Gup intervention groups (L-MG group and H-MG group) exhibited a notable decrease in the FBG compared to the MC group (*p* < 0.05). Remarkably, similar trends were also observed in the area under the curve (AUC) of the OGTT (Figure 2c), incremental AUC (Figure 2d) and HOMA-IR (Figure 2e). Following the intervention, the MN-Gup intervention groups showed a significant reduction in the AUC of the OGTT (*p* < 0.05) and HOMA-IR (*p* < 0.05) compared to the MC group. There were no significant differences in the AUC of the OGTT and HOMA-IR among the MF group, L-MG group, and H-MG group after intervention. In addition, we found no significant differences in insulin among the groups (Figure S1a). However, concerning the FBG levels, the MF group demonstrated significantly lower levels compared to the L-MG group post-intervention (*p* < 0.05), while no significant difference was observed between the MF group and the H-MG group. In summary, these findings suggest that both low-dose and high-dose MN-Gup exhibit promising hypoglycemic and insulin resistance-alleviating effects. Furthermore, the high-dose MN-Gup group exhibited a more pronounced reduction in fasting glucose levels compared to the low-dose MN-Gup group, demonstrating a similarity to the positive control drug metformin. This observation suggests a potential dose-dependent relationship of MN-Gup.

### 4.2. Effects of MN-Gup on Colon and Pancreatic Tissue in T2DM Mice

**Figure 3:**
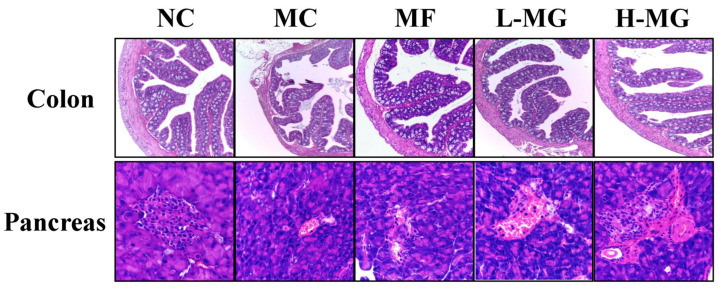
Hematoxylin and eosin (H & E) staining of colon and pancreas tissues of T2DM mice. NC: normal control; MC: diabetic model control; MF: metformin; L-MG: low-dose MN-Gup; H-MG: high-dose MN-Gup.

We employed a histopathological examination of colon and pancreatic tissues (H & E staining) to analyze the degree of lesions (Figure 3). In the NC group, the colonic epithelium and mucosal architecture remained intact. Conversely, the MC group exhibited partial epithelial cell damage and sloughing. However, both the L-MG and H-MG groups demonstrated colonic epithelial and mucosal structures akin to those of the NC group. The pancreatic tissue findings mirrored those observed in the colon. In the NC group, the pancreatic islet structure of mice remained intact, with a plentiful and evenly distributed population of islet cells. Conversely, in the MC group, severe atrophy of the pancreatic islet tissue was observed, accompanied by a reduction in islet area. However, in both the L-MG and H-MG groups, there was a mitigation of pancreatic tissue pathology. There was an increase in the number of islet cells, indicative of restoration.

### 4.3. Effects of MN-Gup on Gut Microbiota Composition and Structure in T2DM Mice

**Figure 4:**
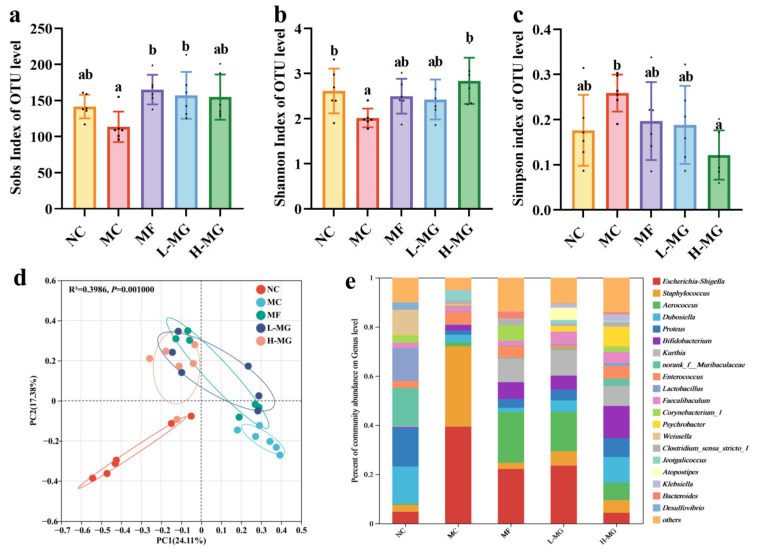
Effect of probiotic MN-Gup on gut microbiota composition in T2DM mice. (**a**) Shannon index of different groups at OTU level. (**b**) Simpson index of different groups at OTU level. (**c**) Sobs index of different groups at OTU level. (**d**) Principal coordinates analysis (PCoA) of the different groups at the OUT level. (**e**) Percent of community abundance on genus level. The sample size for each group was 6. Significance analysis was performed using a one-way ANOVA or the Kruskal–Wallis test, with multiple comparisons conducted using Tukey’s test or Dunn’s test, respectively. The letters a and b represent the results of multiple comparisons. Different letters signify significant differences between groups (*p* < 0.05), while groups sharing the same letter indicate no significant difference. NC: normal control; MC: diabetic model control; MF: metformin; L-MG: low-dose MN-Gup; H-MG: high-dose MN-Gup.

Fecal samples from the T2DM mice were MiSeq sequenced, then clustered for 97% similarity and annotated using the silva132/16s_bacteria database. The results of the diversity analysis are illustrated in Figure 4a–c. The Shannon and Sobs indices of the H-MG group exhibited a marked increase in comparison to those of the MC group (*p* < 0.05). Conversely, the Simpson index for the H-MG group demonstrated a significant decrease relative to that of the MC group (*p* < 0.05). A similar trend was evident in the L-MG group, where the Sobs index was notably higher than that in the MC group (*p* < 0.05). Although no significant difference was observed between the MC group and the L-MG group regarding the Shannon and Simpson indices, their trends aligned with those of the H-MG group.

The bacterial community composition of different groups was studied by PCoA analysis based on the Bray–Curtis distance (Figure 4d). The results showed a clear separation among groups (R^2^ = 0.3986, *p* = 0.001, PC1 = 24.11%, PC2 = 17.38%). The confidence ellipses of the L-MG group and H-MG group overlapped, indicating that these two bacterial communities had similarities. Comparing each group separately, it was observed that the confidence ovals of the NC group (Figure S2a, R^2^ = 0.4348, *p* = 0.003, PC1 = 48.71%, PC2 = 17.82%), L-MG group (Figure S2b, R^2^ = 0.301, *p* = 0.003, PC1 = 44.43%, PC2 = 24.19%), and H-MG group (Figure S2c, R^2^ = 0.3849, *p* = 0.003, PC1 = 43.10%, PC2 = 19.41%) did not overlap with those of the MC group. However, the confidence ellipses of the L-MG group and the H-MG group overlapped (Figure S2d, R^2^ = 0.1506, *p* = 0.061, PC1 = 28.55%, PC2 = 23.30%), indicating that these two bacterial communities had similarities.

**Figure 5:**
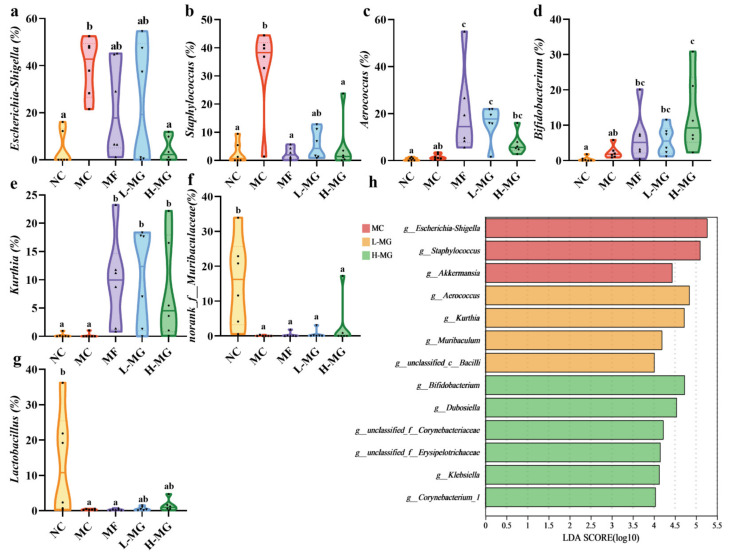
Results of species difference analysis of gut microbiota in T2DM mice. (**a**) Relative abundance of *Escherichia-Shigella*. (**b**) Relative abundance of *Staphylococcus*. (**c**) Relative abundance of *Aerococcus*. (**d**) Relative abundance of *Bifidobacterium*. (**e**) Relative abundance of *Kurthia*. (**f**) Relative abundance of *norank_f__Muribaculaceae*. (**g**) Relative abundance of *Lactobacillus*. (**h**) Linear discriminant analysis effect size (LEfSe) analysis of gut microbiota in T2DM mice. The sample size for each group was 6. Significance analysis was performed using a one-way ANOVA or the Kruskal–Wallis test, with multiple comparisons conducted using Tukey’s test or Dunn’s test, respectively. The letters a, b, and c represent the results of multiple comparisons. Different letters signify significant differences between groups (*p* < 0.05), while groups sharing the same letter indicate no significant difference. NC: normal control; MC: diabetic model control; MF: metformin; L-MG: low-dose MN-Gup; H-MG: high-dose MN-Gup.

Species annotation results revealed that the predominant phyla were *Firmicutes*, *Proteobacteria*, *Actinobacteria*, and *Bacteroidota* (Figure S3). The analysis of microbiota composition at the genus level showed that the top ten species were *Escherichia-Shigella*, *Staphylococcus*, *Aerococcus*, *Dubosiella*, *Proteus*, *Bifidobacterium*, *Kurthia*, *norank_f__Muribaculaceae*, *Enterococcus*, and *Lactobacillus* (Figure 5e). After six weeks of MN-Gup intervention, some bacterial abundances changed. Among the top 10 strains in relative abundance, seven strains showed significant differences among groups. *Bifidobacterium* increased in the L-MG group and H-MG group (Figure 5d). The abundance of *Bifidobacterium* in the H-MG group was notably higher compared to that in the MC group (*p* < 0.05). In terms of the mean relative abundance of the strains, the relative abundance of *Bifidobacterium* doubled in the L-MG group and tripled in the H-MG group compared to the MC group. However, the relative abundance of *Escherichia-Shigella* (Figure 5a) and *Staphylococcus* (Figure 5b) in the H-MG group was significantly lower than that in the MC group (*p* < 0.05). Furthermore, the abundance of *Aerococcus* (Figure 5c) and *Kurthia* (Figure 5e) exhibited higher levels in the MF, L-MG, and H-MG groups, while they were less abundant in the NC and MC groups. Conversely, the relative abundance of *norank_f__Muribaculaceae* (Figure 5f) and *Lactobacillus* (Figure 5g) in the NC group was notably higher than that in the other groups.

Moreover, linear discriminant analysis effect size (LEfSe) can help to find good biomarkers of microbial communities [16]. LEfSe analysis (Figure 5h) showed the MC group was significantly enriched with *Escherichia-Shigella* and *Staphylococcus*. Consistent with the results of previous strain analysis, *Aerococcus* and *Kurthia* were markedly enriched in the L-MG group, whereas *Bifidobacterium* was significantly enriched in the H-MG group.

### 4.4. Effect of MN-Gup on the Composition of SCFAs and GLP-1 in T2DM Mice

**Figure 6:**
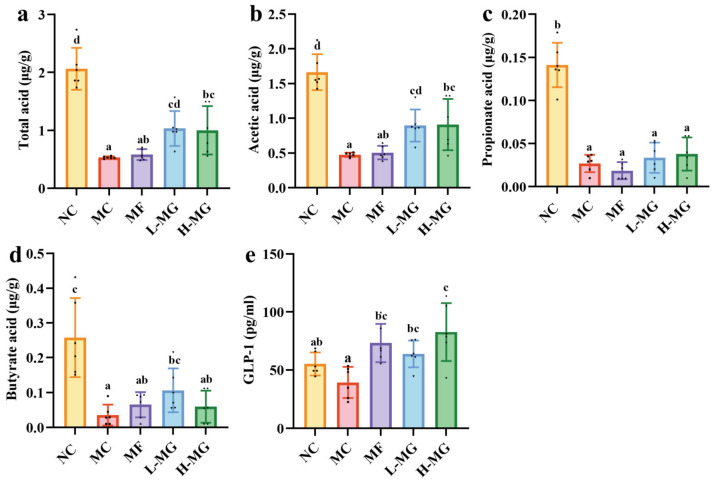
Effect of MN-Gup on the composition of short-chain fatty acids (SCFAs) and glucagon-like peptide-1 (GLP-1) in T2DM mice. (**a**) Fecal total acid concentration in T2DM mice. (**b**) Fecal acetic acid concentration in T2DM mice. (**c**) Fecal propionate acid concentration in T2DM mice. (**d**) Fecal butyrate acid concentration in T2DM mice. (**e**) Effect of MN-Gup on serum GLP-1 in T2DM mice. The sample size for each group was 6. Significance analysis was performed using a one-way ANOVA or the Kruskal–Wallis test, with multiple comparisons conducted using Tukey’s test or Dunn’s test, respectively. The letters a, b, c, and d represent the results of multiple comparisons. Different letters signify significant differences between groups (*p* < 0.05), while groups sharing the same letter indicate no significant difference. NC: normal control; MC: diabetic model control; MF: metformin; L-MG: low-dose MN-Gup; H-MG: high-dose MN-Gup.

Gut microbiota-derived metabolites, such as short-chain fatty acids (SCFAs), are recognized as pivotal molecular mediators, bridging the interaction between the microbiota and the host [17]. To investigate the potential mechanism of intestinal flora intervention in attenuating T2DM induced by a high-fat diet and STZ, we sought to analyze the levels of SCFAs in the fecal samples. As shown in Figure 6, in terms of total acids (Figure 6a), both the L-MG group and H-MG group exhibited significantly higher levels compared to the MC group (*p* < 0.05). Specifically, acetic acid (Figure 6b) was notably elevated in these two groups compared to the MC group (*p* < 0.05), while propionic acid (Figure 6c) and butyric acid (Figure 6d) levels in the L-MG group and H-MG group did not show significant differences compared to those in the MC group.

Given the well-established role of SCFAs in mediating gut microbiota–host interactions, and building upon the analysis of SCFAs, we now redirect our attention to GLP-1, a pivotal incretin hormone intricately involved in the maintenance of glucose homeostasis [18]. As shown in Figure 6e, GLP-1 levels were notably elevated in both the L-MG and H-MG groups compared to the MC group (*p* < 0.05). Furthermore, there was no significant difference in the GLP-1 levels between the L-MG and H-MG groups. However, the mean GLP-1 level in the H-MG group was higher than that in the L-MG group.

### 4.5. Correlation between Gut Microbiota and Environmental Factors in T2DM Mice

**Figure 7:**
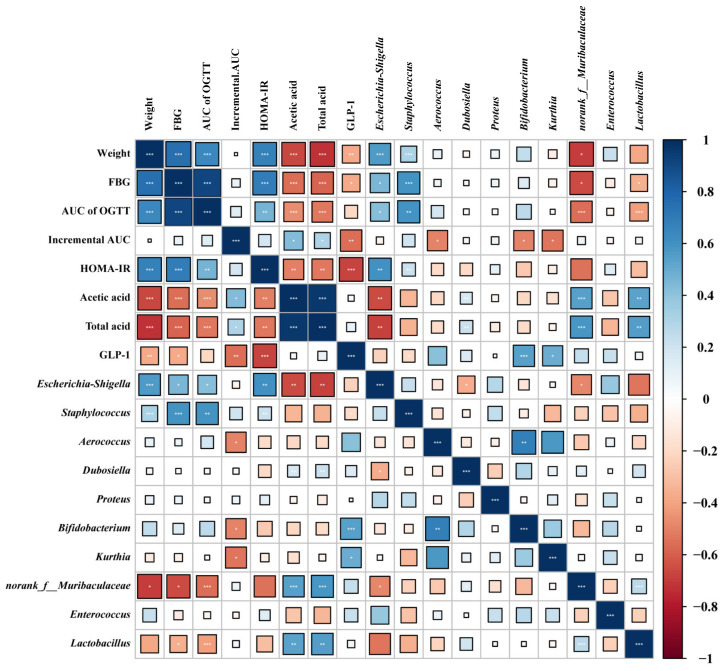
Correlation heat map of gut microbiota and metabolic parameters in T2DM mice. The correlation coefficient matrix was calculated using Spearman’s method. All groups (five groups, a total of 30 mice) were included in the analysis. The significance test of correlation coefficients is denoted as follows: * *p* < 0.05, ** *p* < 0.01, and *** *p* < 0.001.

A correlation analysis can reveal the correlation among complex factors [19]. Correlation heat maps were used to analyze the relationship between the top ten abundant species of different environmental factors at the genus level (Figure 7). Firstly, we observed a significant negative correlation between the relative abundances of *Escherichia-Shigella* and *Staphylococcus* and FBG, the AUC of the OGTT, and HOMA-IR. Conversely, the relative abundances of *Lactobacillus* exhibited a negative correlation with the FBG and the AUC of the OGTT. Secondly, we noted a positive correlation between the relative abundances of *Dubosiella* and *Lactobacillus* and the total acid content and the acetic acid content. Additionally, the relative abundance of *Bifidobacterium* demonstrated a significant positive correlation with the GLP-1 content, which in turn exhibited negative correlations with the incremental AUC.

## 5. Discussion

Given the alarming prevalence, profound health risks, and substantial economic burden associated with T2DM [2], the quest for pragmatic and efficacious intervention strategies to mitigate its impact on public health and societal development is paramount. An accumulating body of evidence underscores the pivotal role of the gut microbiota in the etiology and progression of T2DM [20]. Among the intervention modalities for diabetes, the incorporation of probiotics into dietary regimens to modulate the gut microbiota, thus orchestrating hormone secretion and metabolic homeostasis, has emerged as a burgeoning area of research inquiry [21]. In this study, we observed that *Bifidobacterium animalis* subsp. *lactis* MN-Gup demonstrates notable hypoglycemic effects in mice with T2DM. Additionally, MN-Gup is capable of modulating the gut microbiota of mice and promoting the secretion of SCFAs and GLP-1.

The establishment of animal models is crucial for ensuring the accuracy and reliability of experimental results, as it enables a better simulation of the human disease process and facilitates the evaluation of treatment efficacy and safety. In the progression of T2DM, insulin resistance is predominantly instigated by environmental factors (such as a high-calorie and nutritionally imbalanced diet, deficiency in incretin secretion, etc.) [22]. Subsequently, there is a decline in the insulin secretion function and an impairment of the islet beta cell function, ultimately resulting in inadequate insulin secretion [23]. Therefore, we opted for a high-fat diet to induce insulin resistance in an animal model [24]. We utilized streptozotocin (STZ) to specifically damage pancreatic beta cells, resulting in inadequate insulin secretion [25]. We chose C57BL/6J male mice, known for their sensitivity to STZ, to establish our T2DM model [24,26].

In this study, we evaluated the hypoglycemic effect of MN-Gup in mice, focusing on body weight, blood glucose levels, and HOMA-IR as primary indicators. The MN-Gup treatment resulted in weight loss, consistent with previous findings [15,27]. Compared to the control group, both low and high doses of MN-Gup significantly reduced the FBG levels (L-MG: MD = −3.13 mmol/L, CI [−4.87, −1.40] mmol/L; H-MG: MD = −3.83 mmol/L, CI [−5.56, −2.10] mmol/L, *p* < 0.001, Table S1). A meta-analysis of *Bifidobacterium* probiotics indicated MN-Gup’s superior hypoglycemic effect compared to other strains [28]. Additionally, MN-Gup exhibited efficacy comparable to metformin, a first-line therapy for T2DM [29], with reductions in the FBG levels (MF: MD = −5.02 mmol/L, CI [−6.75, −3.28] mmol/L, *p* < 0.001, Table S1). MN-Gup also improved glucose tolerance, as indicated by the OGTT results. Moreover, both low and high doses of MN-Gup significantly reduced the HOMA-IR levels compared to the control group (*p* < 0.05, Table S1), suggesting an amelioration of insulin resistance. These findings suggest that MN-Gup effectively ameliorates hyperglycemic symptoms and mitigates insulin resistance in T2DM mice, showing superior efficacy compared to other *Bifidobacterium* strains and demonstrating a dose-dependent relationship.

Building upon the impressive phenotypic results, there is a compelling need to delve deeper into the underlying mechanisms. Advancements in next-generation sequencing of 16S rRNA gene amplicons have enabled the acquisition of detailed taxonomic information, particularly at the genus level, regarding the mouse gut microbiota [30]. Notably, alterations in gut microbiota composition have been implicated in T2DM, with increased *Proteobacteria* abundance and decreased diversity commonly observed in T2DM patients [31,32]. Consistent with prior findings, our PCoA and phylum-level analysis corroborate these trends, underscoring the validity of our T2DM mouse model. Upon intervention with MN-Gup, significant improvements in the gut microbiota diversity and evenness were evident in T2DM mice. Moreover, MN-Gup intervention led to a notable reduction in *Proteobacteria* abundance, indicative of microbiota balance restoration. At the genus level, MN-Gup administration notably increased the beneficial taxa, such as *Bifidobacterium*, known SCFAs producers that are crucial for intestinal homeostasis [33,34]. Although slight increases in potentially pathogenic genera such as *Aerococcus* and *Kurthia* were observed post-MN-Gup treatment [35,36], correlation analyses revealed minimal association with the phenotypic outcomes, suggesting an overall microbiome stability. Notably, reductions in harmful bacteria such as *Escherichia-Shigella* and *Staphylococcus* correlated with decreased blood glucose levels [37,38], aligning with our results [39,40]. In summary, MN-Gup intervention effectively modulated the gut microbiota composition in T2DM mice, promoting beneficial taxa, while reducing the harmful ones, thus contributing to improved metabolic parameters.

Furthermore, metabolites derived from the gut microbiome are crucial factors in T2DM and are intricately linked to the composition of the gut microbiota [41]. Among these metabolites, SCFAs are some of the most common and widely studied metabolites of the gut microbiota [4]. SCFAs, comprising organic acids, are produced through the fermentation of dietary fiber by gut microbiota in the large intestine [17]. Acetic acid, propionate acid, and butyrate acid are the three main types of SCFAs. They have been reported to play multiple roles in T2DM, including promoting intestinal epithelial integrity, as well as regulating pancreatic β-cell proliferation, GLP-1, and insulin secretion [42]. Consistent with previous results [43,44], we also observed an increase in the SCFA content (especially acetic acid), accompanied by an increase in GLP-1. This is consistent with our finding, suggesting that SCFAs may exert their influence on T2DM by increasing GLP-1 secretion. GLP-1 is an essential hormone secreted by intestinal endocrine cells, playing a pivotal role in regulating insulin secretion, insulin resistance, and appetite regulation [45]. Based on the results of the correlation analysis and previous research, we speculate that MN-Gup may contribute significantly to hypoglycemic effects by modulating gut microbiota and promoting the secretion of SCFAs and GLP-1.

## 6. Conclusions

In summary, our research demonstrates that *Bifidobacterium animalis* subsp. *lactis* MN-Gup has significant hypoglycemic effects in T2DM mice, and can modulate the gut microbiota, promoting the secretion of SCFAs and GLP-1. MN-Gup effectively ameliorates hyperglycemic symptoms and mitigates insulin resistance in T2DM mice, showing superior efficacy compared to other *Bifidobacterium* strains and demonstrating a dose-dependent relationship. This research sheds light on the critical role of the gut microbiota in the pathophysiology of T2DM, providing a solid foundation for future studies aimed at developing precision probiotic treatments.

## Data Availability

The Supplementary Materials File, Origin Data.xlsx, contains detailed data sets and additional information related to the findings presented in this study. Additional inquiries should be addressed to the corresponding author.

## Supplementary Materials

The following supporting information can be downloaded at: https://www.mdpi.com/article/10.3390/nu16111691/s1, Figure S1: Effect of MN-Gup on fasting insulin and blood glucose in T2DM mice. Figure S2: Principal coordinates analysis (PCoA) analysis of different groups at the OUT level. Figure S3: Circos Representation of Phylum-Level Gut Microbiota in Mice. Table S1: Effect of MN-Gup intervention on glucose metabolism indices.
