# Supplementary material for: The Effect of Bifidobacterium animalis subsp. lactis MN-Gup on Glucose Metabolism, Gut Microbiota, and Their Metabolites in Type 2 Diabetic Mice
Source: Nutrients. 2024 May 29;16(11):1691. doi: 10.3390/nu16111691 (PMC11174421; doi:10.3390/nu16111691)
Supplement: Supplementary file 1 [file nutrients-16-01691-s001.zip › Supplementary material.docx]

# Supplementary material


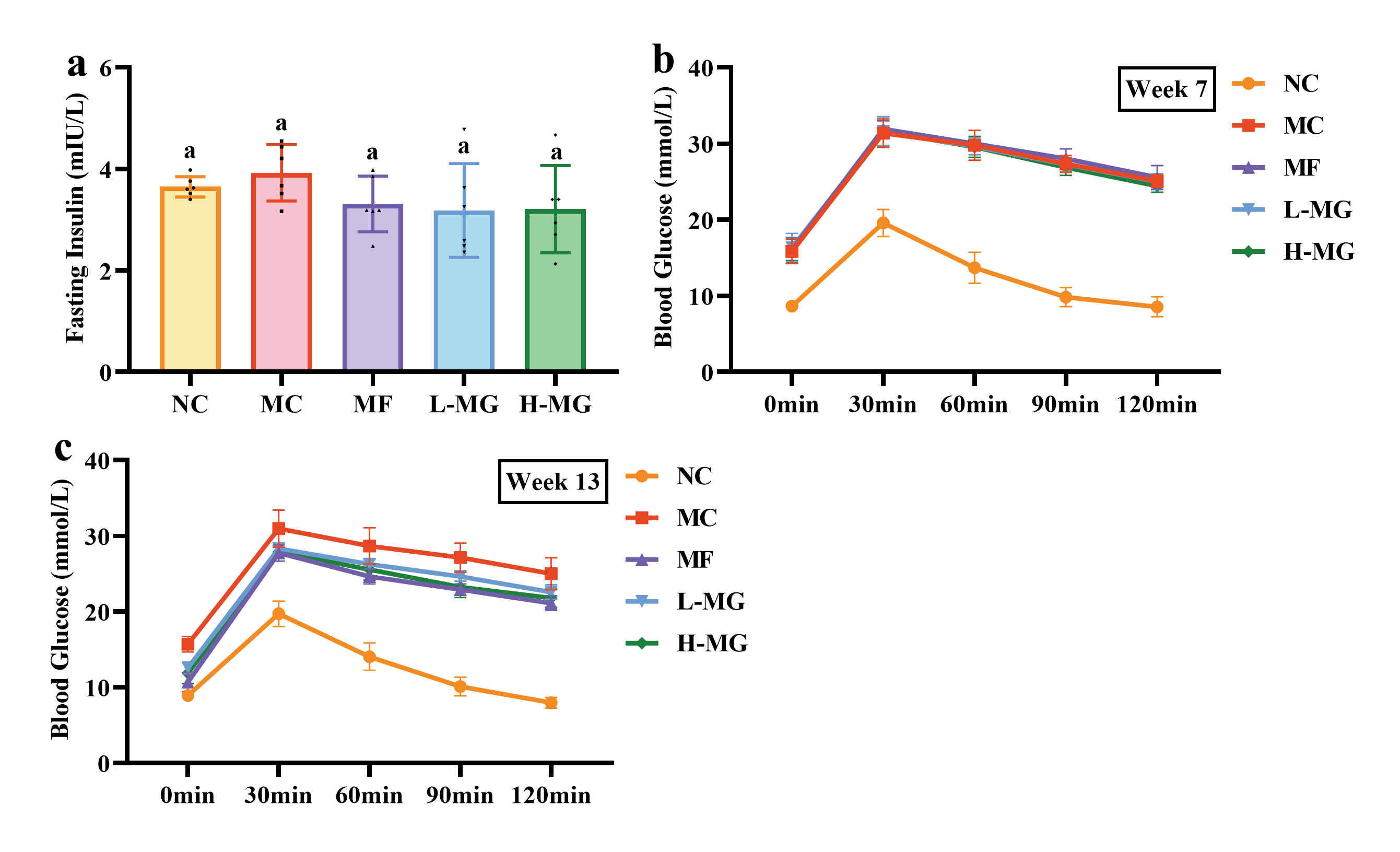


**Supplementary Figure 1** Effect of MN-Gup on fasting insulin and blood glucose in T2DM mice. (**a**) Fasting insulin level at week 13. (**b**) Blood glucose level at week 7. (**c**) Blood glucose level at week 13. The sample size for each group was 6. Significance analysis was performed using one-way ANOVA or Kruskal-Wallis test, with multiple comparisons conducted using Tukey's test or Dunn's test, respectively. The letters a represent the results of multiple comparisons. Groups sharing the same letter indicate no significant difference. NC: normal control; MC: diabetic model control; MF: metformin; L-MG: low dose MN-Gup; H-MG: high dose MN-Gup.


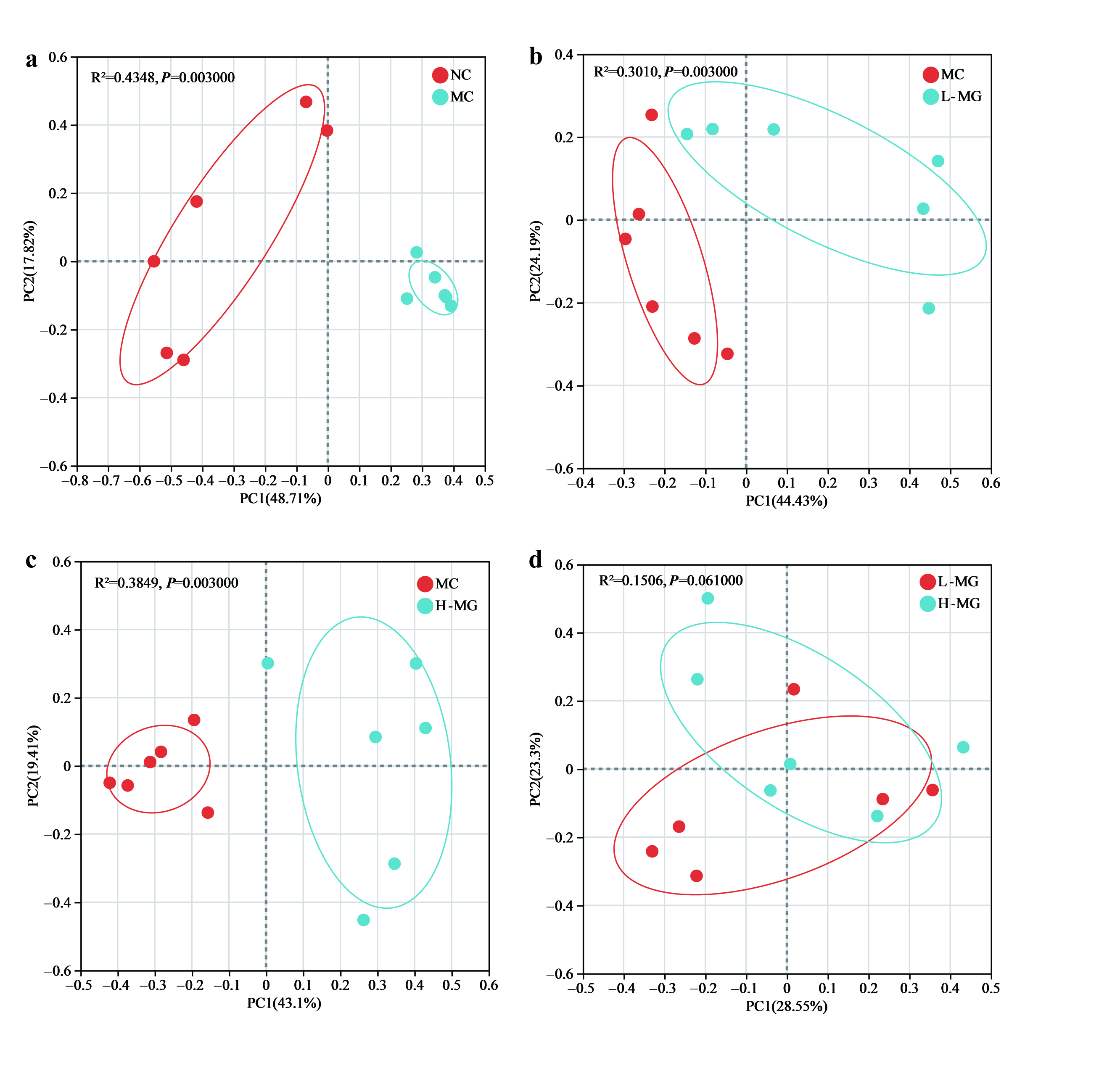


**Supplementary Figure 2** Principal coordinates analysis (PCoA) analysis of different groups at the OUT level. (**a**) PCoA analysis between NC group and MC group. (**b**) PCoA analysis between the NC group and L-MG group. (**c**) PCoA analysis between the MC group and H-MG group. (**d**) PCoA analysis between L-MG group and H-MG group. NC: normal control; MC: diabetic model control; L-MG: low dose MN-Gup; H-MG: high dose MN-Gup.


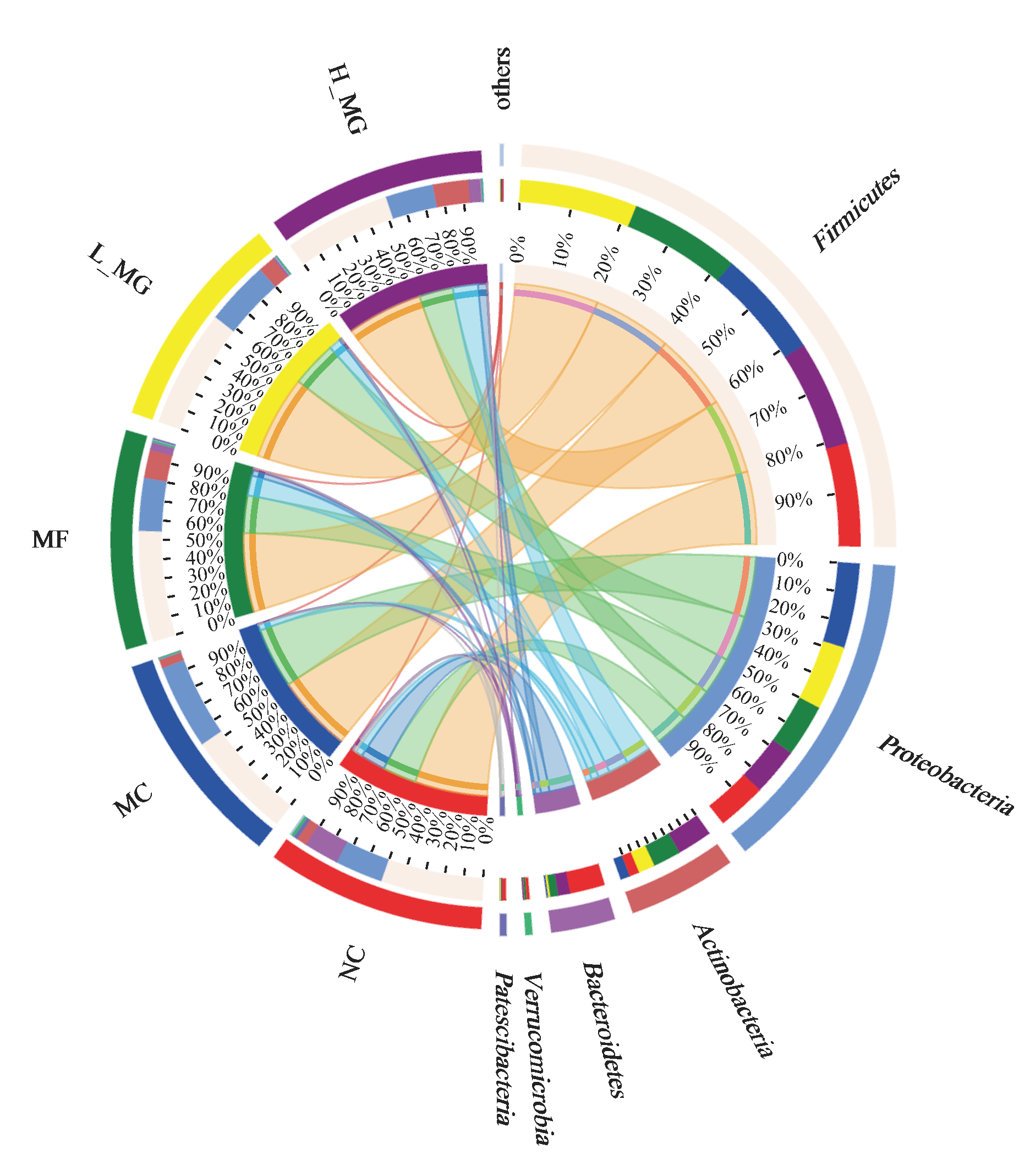


**Supplementary Figure 3** Circos Representation of Phylum-Level Gut Microbiota in Mice. NC: normal control; MC: diabetic model control; MF: metformin; L-MG: low dose MN-Gup; H-MG: high dose MN-Gup.

**Supplementary Table 1** Effect of MN-Gup intervention on glucose metabolism indices

| **Variables** | **Groups** | **n** | **Results** | **Differennce (95%CI)** | ***P_1_*** | ***P_2_*** |
| --- | --- | --- | --- | --- | --- | --- |
| **FBG (week 13)** | **NC** | 6 | 8.92±0.29 | -6.78 [-8.52, -5.05] | <0.001 | <0.001 |
|  | **MC** | 6 | 15.70±1.01 | / |  | / |
|  | **MF** | 6 | 10.68±1.24 | -5.02 [-6.75, -3.28] |  | <0.001 |
|  | **L-MG** | 6 | 12.57±0.78 | -3.13 [-4.87, -1.40] |  | <0.001 |
|  | **H-MG** | 6 | 11.87±1.40 | -3.83 [-5.56, -2.10] |  | <0.001 |
| **HOMA-IR (week 13)** | **NC** | 6 | 1.42 (1.37, 1.55) | / | 0.017 | 0.002 |
|  | **MC** | 6 | 2.75 (2.40, 3.03) |  |  | / |
|  | **MF** | 6 | 1.67 (1.39, 1.76) |  |  | 0.015 |
|  | **L-MG** | 6 | 1.57 (1.33, 2.28) |  |  | 0.017 |
|  | **H-MG** | 6 | 1.50 (1.27, 2.20) |  |  | 0.006 |

Difference: The confidence interval for the difference in group effect values, with the MC group as the control group; CI: Confidence interval; FBG: Fasting blood glucose; HOMA-IR: Homeostatic Model Assessment for Insulin Resistance; NC: normal control; MC: diabetic model control; MF: metformin; L-MG: low dose MN-Gup; H-MG: high dose MN-Gup; ***P_1_***: *P*-Value for One-Way ANOVA or Kruskal-Wallis Test Among 5 Groups; ***P_2_***: P-Values of Multiple Comparisons Using Tukey's Method or Dunn's Method, with the MC Group as the Control.
